# Trajectories and Predictors of Change in Emotion Dysregulation and Deliberate Self-Harm Amongst Adolescents with Borderline Features

**DOI:** 10.1177/13591045231177374

**Published:** 2023-05-23

**Authors:** Iselin Solerød Dibaj, Anita Johanna Tørmoen, Ole Klungsøyr, Egil Haga, Lars Mehlum

**Affiliations:** Institute of Clinical Medicine, University of Oslo, 150754 National Center for Suicide Research and Prevention, Oslo, Norway

**Keywords:** deliberate self-harm, adolescence, emotion regulation, psychotherapy, suicidality

## Abstract

**Background:**

Deliberate self-harm (DSH) and emotion dysregulation (ED) peaks in adolescence, and is associated with an increased risk of psychopathology, suicide and lower functioning in adulthood. DBT-A has been established as an effective treatment for reducing DSH, however less is known about changes in emotion dysregulation. This study aimed to identify baseline predictors of treatment response in outcome trajectories of DSH and emotion dysregulation.

**Methods:**

Response trajectories of DSH and ED were investigated using Latent Class Analysis on RCT data comparing DBT-A and EUC for 77 adolescents treated for deliberate self-harm and borderline traits. Logistic regression analysis was used to examine baseline predictors.

**Results:**

Two-class solutions were selected for both indicators, distinguishing between early and late responders in DSH, and responders and non-responders in ED. Higher levels of depression, shorter DSH histories and not receiving DBT-A predicted less favourable response in DSH, while DBT-A was the only predictor of treatment response in ED.

**Conclusions:**

DBT-A was associated with a significantly faster reduction of deliberate self-harm in the short-term and improved emotion regulation in the long-term.

## Introduction

Emotion dysregulation (ED), the inability to flexibly respond to and manage emotions (Carpenter & Trull, 2013), and recurrent deliberate self-harm [DSH], defined as nonfatal self-poisoning or self-injury with or without suicidal intent (Hawton et al., 2002), are core features of Borderline Personality Disorder [BPD]. Both emotional dysregulation and self-harm peak in adolescence (Plener et al., 2015; Compas et al., 2017), and a growing recognition of this disorder’s validity in adolescents has corresponded to an increase in clinical trials for BPD youth (Wong et al., 2020). Specialized interventions for adolescents with BPD, such as Dialectical Behaviour Therapy [DBT-A] (Mehlum et al., 2014; McCauley et al., 2018) and Mentalization Based Therapy (Rossouw & Fonagy, 2012; Beck et al., 2020), have shown that it is possible to reduce self-harm and suicidal ideation for this group.

DBT-A is the treatment with strongest empirical support for its effectiveness in treatment of adolescents with BPD, with three randomized controlled trials (Mehlum et al., 2014; McCauley et al., 2018; Santamarina-Perez et al., 2020) and a systematic review (NHMRC, 2009; Kothgassner et al., 2021), and was recently included in the NICE guidelines for emotionally dysregulated young people with repeated self-harm (NICE, 2022). Still, we lack knowledge on response trajectories and what characterize patients who respond to DBT-A; which could help improve treatment-patient matching and refine interventions. A systematic review found that therapeutic alliance and higher symptom severity predicted superior outcomes across adult BPD clinical trials (Barnicot et al., 2012). Using Latent Class Analysis (LCA) to explore change trajectories and patient-related predictors, studies have found higher levels of baseline depression, unemployment and emergency room visits to be associated with less favourable outcomes in adult DBT (McMain et al., 2018), and that re-hospitalization predicted reduced suicidal ideation among adolescents, however only when baseline suicidal ideation was high (Czyz et al., 2016). To our knowledge, two studies have used LCA on RCT data with suicidal adolescents: First, higher self-reported depression, pessimism and self-harm predicted non-response in depression and suicidal ideation in Attachment-Based Family-Therapy (Abbott et al., 2019). Second, DSH non-response was associated with more baseline nightmares, self-reported depression and parent-reported externalizing in DBT-A (Berk et al., 2022). However, there is a scarcity of studies of how emotion dysregulation changes during therapy for adolescents, and how this relates to changes in DSH.

As ED and DSH are core symptoms in adolescent BPD, it is important to increase our knowledge of how these factors change after treatment. Several studies support emotion regulation as a mechanism of change in DBT’s effect on DSH in adults (Barnicot et al., 2016; Axelrod et al., 2011). In DBT, improved emotion regulation is assumed to be responsible for reduced DSH, through acquisition, practice and generalization of effective emotion regulation skills (Linehan, 1993). Moreover, the reason for targeting DSH in DBT is twofold. Firstly, it is a [maladaptive] emotion regulation strategy that maintains and exacerbates emotion dysregulation (Houben et al., 2017). Secondly, it is a problem behaviour linked to high risks of serious outcomes such as suicide attempts (Asarnow et al., 2011) and completed suicide (Olfson et al., 2017). Not unexpectedly, BPD patients tend to use some emotion regulation skills naturally before engaging in treatment (Stanley et al., 2021), and an increase in skills use during DBT is a mediator of DSH reduction (Neacsiu et al., 2010, 2014). Moreover, increased use of skills was associated with less drop-out and DSH reduction independently of therapeutic alliance (Barnicot et al., 2016). Similarly, DSH reductions were mediated by improved emotion regulation and partially mediated by increased skills use, in adolescents receiving DBT-A (Asarnow et al., 2021). Finally, emotion regulation improvements mediated DSH reductions in adolescents receiving Cognitive Behavioural Therapy (Slee et al., 2008) and Emotion Regulation Individual Therapy for Adolescents (Bjureberg, 2022).

Although DSH can serve various functions, the most common is to regulate emotions (Kuehn et al., 2022). In the short term, DSH reduces negative affect (Rodriguez-Blanco et al., 2018), however reinforces long-term dysregulated affect (Nock & Prinstein, 2004). Earlier age of onset and longer history of DSH predicted higher DSH frequency and increased risk of suicide attempts in treatment-seeking adolescents (Brager-Larsen et al., 2022). Additionally, repeated DSH has been related to increased inclination to act on suicidal urges through pain habituation and diminished fear of death (Hamza et al., 2012; Van Orden et al., 2010). Taken together, DSH is an important treatment outcome, both directly and indirectly. Directly, as reductions in DSH implies reductions in a dangerous problem behaviour associated with increased suicide risk – and indirectly, as such reductions might reflect improved emotion regulation, which is in turn associated with reduced risk of psychopathology, improved functioning and quality of life (Young et al., 2019). Thus, it can be expected that DSH reduction might predict subsequent improvements in emotion regulation; however, this relationship remains unclear. Where earlier DBT studies of response trajectories have focused mostly on suicidal ideation and self-harm (McMain et al., 2018; Berk et al., 2022), less is known about other core BPD symptoms such as emotion dysregulation.

The present study aimed to explore patterns of deliberate self-harm and emotion dysregulation; how these changed after treatment, and whether DSH reduction corresponded to subsequent emotion regulation improvement in adolescents treated with either DBT-A or enhanced usual care (EUC). Another aim was to investigate potential baseline predictors of change in emotion dysregulation and deliberate self-harm. We expected to identify different response trajectories, and that there would be overlap between responders in DSH and ED. Finally, we hypothesized that non-responders would have more depressive symptoms, earlier age of DSH onset and longer duration of DSH. All analyses included the sample as a whole, and treatment condition was considered in the prediction model.

## Methods

In this study, we performed secondary analyses on data from an RCT comparing DBT-A with EUC.

### Participants and Procedures

The procedures used in this study adhere to the tenets of the Declaration of Helsinki. The study was approved by the Regional Committee for Medical Research Ethics, South-East Norway (Mehlum et al., 2014), and all patients and parents provided written informed consent. Detailed information regarding the trial methods is published elsewhere (Mehlum et al., 2014), but, briefly, participants were included from child and adolescent psychiatric outpatient clinics in Oslo if they had a history of ≥2 episodes of self-harm, wherein ≥1 within the last 16 weeks, ≥2 criteria of DSM-IV BPD (and the self-destructive criterion) *or* ≥1 criterion of DSM-IV BPD *and* ≥2 subthreshold-lever criteria, and Norwegian fluency. Exclusion criteria were a diagnosis of Bipolar I, any psychotic disorder, intellectual disability or Asperger’s syndrome. 294 patients were screened for eligibility, 97 went through clinical assessment and the final sample consisted of 77 adolescents, aged 12–18 years who were then randomized to 19 weeks of either DBT-A or EUC. Baseline assessments were conducted by two child and adolescent psychiatrists and two doctoral level clinicians and tested for interrater reliability. Further assessments occurred at 9, 15 and 19 weeks after treatment start as well as 1.6 and 3.1years post-randomization. The mean time between randomization and post-treatment assessments were 31 weeks or 0.6 years. Randomization was done after baseline assessments, and stratified on gender, presence of major depression and presence of suicide intent during the most serious self-harm episode within the last 16 weeks before study enrolment. Sample characteristics are illustrated in Table 1, adapted from the original RCT. In this analysis, we have included data collected at baseline, 19 weeks (only DSH), 1.6 (only DSH) and 3.1 years follow-up. DBT-A was delivered over 19 weeks, in accordance with the manual developed by Miller et al. (2007) with treatment adherence ratings within the adherent range (Mehlum et al., 2014). EUC consisted of 19 weeks of standard non-DBT treatment as it is delivered in the participating child and adolescent outpatient psychiatric clinics, mainly psychodynamic or cognitive behavioural therapy, in combination with psychopharmacological treatment if indicated. EUC was neither manualized nor adherence checked, and therapists could extend treatment beyond the trial time if needed. The enhanced aspect of the treatment was that EUC therapists agreed to deliver at least weekly individual therapy sessions for the duration of the trial, that all therapists received information on results from trial assessments made of their patients and that all therapists received training in suicide risk assessment and management before inclusion of patients commenced.

**Table 1.** Sample Characteristics of Adolescents (*N* = 77) receiving DBT-A or Enhanced Usual Care. Adapted from Mehlum et al., 2014.

Variable	DBT (*n* = 39)		EUC (*n* = 38)		Total sample (*N* = 77)	
	n	%	n	*%*	*n*	*%*
Female sex	34	87.2	34	89.5	68	88.3
Norwegian ethnicity	30	78.9	32	91.4	62	84.9
High school graduate	15	39.5	12	32.4	27	36.0
Parents currently married	17	43.6	17	44.7	34	44.2
Child protection (current)	6	15.4	7	18.4	13	16.9
Child protection (past)	10	26.3	11	28.9	21	27.6
Past psychiatric treatment	28	73.7	23	62.2	51	68.0
Past psychopharmacotherapy	2	5.4	6	17.1	8	11.1
Current psychopharmacotherapy	6	15.4	3	7.9	9	11.7
Current DSM-IV axis I or II diagnosis						
MDD	9	23.1	8	21.1	17	22.1
Other depressive disorder	16	41.0	13	34.2	29	37.7
Panic disorder	2	5.1	5	13.2	7	9.1
PTSD	7	17.9	6	15.8	13	16.9
Any anxiety disorder	18	46.2	15	39.5	33	42.9
Any SUD	1	2.6	1	2.6	2	2.6
Any eating disorder	3	7.7	3	7.9	6	7.8
BPD	10	26.3	5	14.3	15	20.5
Attempted suicide last 4 months	11	28.2	9	23.7	20	26.0
	Mean	SD	Mean	SD	Mean	SD
Age (y)	15.9	1.4	15.3	1.6	15.6	1.5
C-GAS score	55.3	8.0	57.9	10.1	56.1	8.3
CBCL total score, by parents	69.6	11.0	68.4	8.9	69.0	9.8
BPD criteria fulfilled*	4.0	2.0	3.0	3.0	4.0	2.0
Current DSM-IV axis I disorder (*n*)*	2.0	1.0	1.0	3.0	1.0	2.0
Suicide attempt, lifetime (*n*)	2.1	5.2	1.3	2.8	1.7	4.2
Nonsuicidal self-harm, lifetime (*n*)*	49.5	159.5	25.0	45.5	34.0	88.0

Note: BPD = Borderline Personality Disorder; CBCL = Child Behaviour Check List; C-GAS = Children’s Global Assessment Scale; MDD = Major Depressive Disorder; PTSD = Post Traumatic Stress Disorder; SUD = Substance Abuse Disorder. *Median and Interquartile range.

### Variables

The indicator variables in the Latent Class Analysis (LCA) were frequency of deliberate self-harm and emotional dysregulation. DSH frequency was operationalized as self-harm with and without suicide intent and the number of episodes was standardized to number of event per 4-months intervals, and measured by the Lifetime Parasuicide Count (LPC) (Linehan & Comtois, 1996). For ED, we used a constructed proxy variable generated by a sum score (range: 0–10) of the following equally weighted variables: Two BPD diagnostic criteria from SCID-II (First et al., 1997); ‘affective instability’ and ‘inappropriate anger’, the YSR (Achenbach, 1987) items ‘sudden mood swings’ and ‘intense anger’ in addition to the BSL-23 (Bohus et al., 2007) item ‘frequent changes in the mood between anxiety, anger and depression’. This proxy predominately taps into affective features of emotional dysregulation, and has adequate internal consistency (α = .72) (Neupane & Mehlum, 2022). In the baseline predictor analyses, we included baseline self-harm (LPC), age of self-harm onset (LPC), duration of self-harm behaviour and clinician-rated depression (MADRS) (Montgomery & Asberg, 1979). We also investigated associations between class membership and treatment condition.

### Data Analysis

All statistical analyses were conducted in STATA 17.0, and Latent Class Analysis was performed using the STATA LCA plugin developed by Penn State University (The Methodology Center, 2015). First, we used LCA to identify response trajectories in ED and DSH outcomes. The ED model used a Gaussian distribution and included measures at baseline and 3-years follow up. The DSH model used a negative binomial distribution and included measures on baseline, post-treatment, after 1.6 and 3.1 years of follow-up. In the LCA models, maximum-likelihood by the EM-algorithm was used, applying the *missing at random* assumption. We began with the estimation of two-class models and increased the number of classes until convergence was not achieved (Weller et al., 2020). To evaluate goodness-of-fit, we used Bayesian Information Criterion [BIC], Aikaike Information Criterion [AIC] and evaluated parsimony and theoretical meaningfulness. Classes were identified separately across the two indicators, and all participants were assigned class membership to both class solutions, according to their maximum probability of belonging to a certain class based on the probability distributions. Then, we calculated the number of ‘total responders’ and ‘total non-responders’, thus participants that belonged to a responder class in both or neither condition. Finally, we performed univariate and multiple logistic regression analyses to investigate potential baseline characteristics that could predict class membership probability. In the adjusted analyses, we combined the statistically significant single predictors, including treatment condition. Interaction terms were tested and model fit evaluated through likelihood ratio tests, and interaction terms included where indicated. In the ED model, including one interaction term yielded a more robust model. Variables with high multicollinearity were removed.

## Results

### Sample Characteristics

See Table 1 for a summary of the sample characteristics.

### Latent Class Analyses

**Figure 1.**
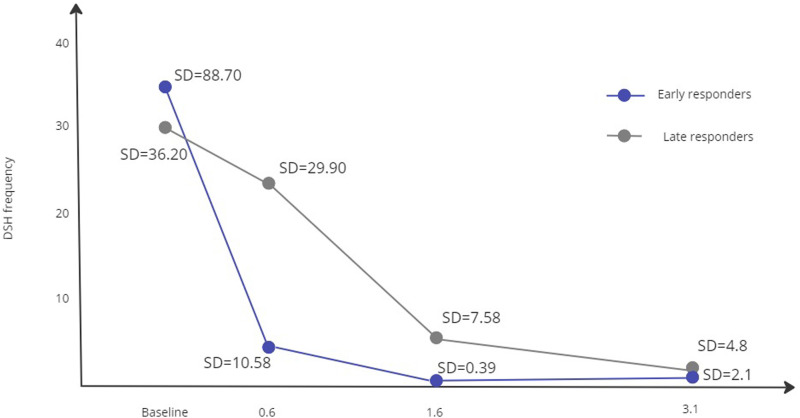
Number of deliberate self-harm (DSH) episodes per 4 months in early vs late responders of adolescents (*N* = 77) receiving DBT-A or Enhanced Usual Care.

**Figure 2.**
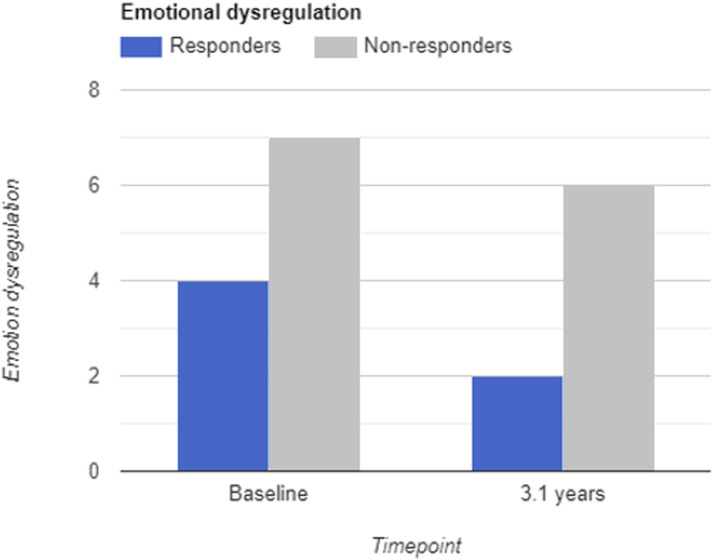
Mean level of emotion dysregulation in responders and non-responders.

The DSH-model yielded up to five classes until convergence was not achieved; for the ED-model the result was four classes (see Table 2). Lower BIC and AIC values indicate better statistical fit, hence, in the DSH analysis, the three-class solution had a slightly better statistical fit. However, it is recommended not to include class solutions that include class(es) with *n* < 5 and since one of the classes had too few cases (*n* = 3) to be meaningful to test statistically, we selected the two-class model. In the ED analysis, the AIC favoured a three-class solution, while the BIC indicated a better fit for the two-class solution. The BIC is generally considered as most useful as it favours parsimony (Weller et al., 2020) which is recommended with smaller sample sizes, and also increases risk of underfitting over overfitting (Dziak et al., 2014). Considering our modest sample size and aim to examine broader differences between responders and non-responders, we selected the two-class model. Response trajectories are presented in Figure 1 and 2.

**Table 2.** “Goodness of fit” statistics for the latent class models.

Model	AIC	BIC
2 classes – DSH	1599.528	1629.998
3 classes – DSH	1587.257	1629.445
4 classes – DSH	1576.711	1630.618
5 classes – DSH	150.670	1646.296
2 classes – ED	647.644	663.8664
3 classes – ED	641.0972	664.2721
4 classes – ED	644.4837	681.5635

Note: AIC = Aikaike Information Criterion, BIC = Bayesian Information Criterion. DSH = Deliberate self-harm, ED = Emotion dysregulation.

There were no differences between classes when it came to DSH frequencies at baseline, however the classes derived from the LCA diverged over time such that one class (52%) was characterized by an earlier and sharper decrease of 85% (thus labelled *early responders*), than the other class (48%) with a less prominent decrease of 24%; (thus labelled *late responders*). Over time, both classes showed large reductions in DSH (98% and 91%, respectively), however early responders showed a higher degree of remission (M = 0.73, SD = 2.1) compared to more variability in late responders (M = 2.8, SD = 4.8). To compare DSH reduction between the classes, we calculated the area under each curve in Figure 1 to represent number of events in the three intervals; baseline to 0.6, 1.6 and 3.1 years later, respectively. The area difference between the two classes was 4681 DSH episodes over the total 3-year period, equivalent to an average of 61 episodes per patient (*N* = 77).

While members of both classes had largely ceased their self-harm behaviour by the 3.1year mark, there were significant differences as to when this remission had occurred: 66% of the late responders reported DSH within the last half of the treatment period, compared to only 18% of the early responders. Mean frequencies of DSH episodes during the treatment phase were 5.20 (SD = 1.69) and 23.11 (SD = 12.68) for early and late responders, respectively. Frequencies and proportions in relation to when participants reported their last self-harm episode are presented in Table 3. Most early responders reported their last DSH episode in the first half of treatment, while most late responders still reported DSH in the final month. Although most (75%) participants that still reported DSH during treatment were late responders, a substantial proportion of late responders (58%) actually reported their last DSH episode during the first 3 months of treatment. Thus, despite achieving less favourable outcomes compared to the early responders, a modest majority actually report DSH cessation during the treatment phase.

**Table 3.** Distribution of last DSH Episode: Differences in Frequencies and Proportions between Response Classes.

Last DSH episode during treatment	Early responders n = 34^a^	Late responders n = 36^a^
	Proportion (cumulative)	Proportion (cumulative)
No DSH during treatment	26% (26%)	3% (3%)
1st month	44% (70%)	31% (34%)
2nd month	12% (82%)	8% (42%)
3rd month	3% (85%)	16% (59%)
Final month	15% (100%)	42% (100%)

^a^*n* = sample size is reduced due to missing data on some participants.

For emotion dysregulation (see Figure 2), there was a notable difference in baseline levels of emotion dysregulation, where one class was characterized by lower mean levels and higher variability (M = 4.68, SD = 2.44) compared to the other (M = 7.56, SD = 1.52). For descriptive purposes, we labelled these classes *responders* (60%) and *non-responders* (40%). Responders showed a mean reduction of two points (39%) compared to a 1-point change in the non-responders (9%). Whereas all non-responders showed high ED baseline levels, responders had a wider range of ED levels at baseline.

Finally, we combined DSH early and late responders with ED responders and non-responders, respectively, to construct broader categories labelled “total responders” and “total non-responders”. When combined, approximately half of the sample was a responder in either both or neither class solution: 14 (19%) was total non-responders compared to 22 (29%) total responders. The trajectories of total responders and non-responders appeared similar to those of responders and non-responders in the respective classes, and the proportions are displayed in Table 4.

**Table 4.** Tabulated Class Membership in Latent Class Analyses of Deliberate Self-Harm and Emotion Dysregulation.

*Deliberate Self-Harm*	Emotion dysregulation		*Total*
	*Non-responder*	*Responder*	
Late responder	14	23	37
Early responder	16	22	38^b^ (40)
Total	30	45	75^b^ (77)

^b^2 of the early responders are excluded from this table due to missing data in the ED model.

### Missing Data

LCA was performed for all participants who started treatment and completed the post-treatment assessment. At the end of treatment, 100% of the original sample participated, 97% at the 1.6year mark (*n* = 75) and 90% (*n* = 70) in the 3.1year mark. Missing data across time points are displayed in Table 5 and was handled using maximum likelihood estimation and specifying the EM algorithm. Based on the LCA, all participants were assigned a DSH class, and all except for two, an ED class.

**Table 5.** Missing data in the indicator variables, by class membership.

*Indicator variable*	Class	Baseline	0.6 years	1.6 years	3.1 years
*Deliberate Self-Harm*	Early responders	3	1	8	10
	Late responders	9	3	2	1
*Emotion Dysregulation*	Responders	1	-	-	3
	Non-responders	4	-	-	5

### Baseline Predictors

Single and combined logistic regressions were performed for DSH late response, ED non-response and total non-response, and are presented in Table 6. Only statistically significant univariate or multivariate predictors are presented.

**Table 6.** Logistic regression analyses of baseline predictors for response class membership.

Variables	Unadjusted			Adjusted		
	OR	p-value	CI: 95%	OR	p-value	CI: 95%
Deliberate self-harm: Late responders						
Treatment condition^c^	2.2	.09	.88 – 5.4	3.83	.015	1.30 – 11.29
MADRS	1.08	.022	1.01 – 1.15	1.09	.015	1.01 – 1.18
DSH duration^d^	.97	.024	.95 – .99	.97	.027	.94 – .99
Emotion dysregulation: Non-responders						
Treatment condition^c^	1.78	.22	.70 – 4.55	7.5	.013	1.53 – 36.77
MADRS	.99	.95	.94 – 1.07	.97	.48	.90 – 1.04
DSH duration^d^	.99	.24	.96 – 1.01	1	.66	.97 – 1.03
Treatment condition X DSH duration	-	-	-	.94	.027	.89 – .99
Total non-response						
Treatment condition^c^	6.41	.018	1.36 – 30.05	10.91	.016	1.55 – 76.70
MADRS	1.07	.12	.97 – 1.18	1.11	.095	.99 – 1.30
DSH duration^d^	.96	.053	.92 – 1.00	.96	.12	.93 – 1.02

^c^EUC,

^d^in months, from first lifetime DSH episode to baseline.

For DSH, higher clinician-rated depression at baseline and shorter DSH duration predicted late treatment response, both adjusted and unadjusted. In the adjusted DSH model, DBT-A was associated with early response. When it comes to ED, the only predictor of treatment response in the adjusted model was allocation to DBT-A. In addition, there was an interaction effect of treatment condition by duration of DSH, where longer duration of DSH in combination with DBT-A predicted non-response. In effect, DBT-A was favourably related to ED response, however not when DSH duration exceeded 41 months. In other words, DSH duration appeared to have a moderating effect for DBT-A on ED. For total non-response, the only statistically significant predictor in both the single and combined model was receiving EUC compared to DBT-A. Baseline DSH frequency and age of DSH onset were not associated with class membership.

## Discussion

This study identified different outcome trajectories in emotion dysregulation and deliberate self-harm in an RCT treating adolescents with borderline features and repetitive deliberate self-harm using a data-driven approach. First, we identified temporal differences in DSH reduction during treatment, and found that DBT-A, lower levels of depressive symptoms and longer DSH history were related to earlier treatment response. Second, our results revealed a subgroup of patients with stable high levels of emotion dysregulation over a 3-year period, compared to another group reporting ED reduction over time. Here, ED reduction was associated with allocation to DBT-A 3 years earlier, however this effect was moderated by DSH duration. Finally, when combining response classes, DBT-A was the only significant predictor for total response; to be a DSH early responder as well as an ED responder. Taken together, these findings indicate that DBT-A not only reduced deliberate self-harm in the short-term, but also led to long-term improvements in emotion regulation.

Our findings are in line with previous research (Plener et al., 2015), showing an overall decrease in DSH from adolescence to early adulthood. Although differences between response classes diminished over time, DBT-A was related to early DSH reduction. Consistently, DBT has been evaluated superior compared to other treatments in DSH reduction, which may be attributed to DBT’s emphasis on emotional dysregulation and changing problem behaviour (such as DSH) through skills training (Linehan, 1993). In DBT, change interventions are balanced with acceptance-based strategies (Carson-Wong et al., 2018), and there is a focus on building a solid therapeutic relationship (Bedics et al., 2012) and a life worth living, which are assumed to reduce drop-out and increase treatment compliance.

Similarly to Berk et al. (2022), we found that higher baseline depression predicted less favourable DSH outcomes. As adolescents in treatment for DSH is a heterogeneous population, this could indicate that adolescents with DSH as part of a depressive disorder rather than a more *generalized* emotion dysregulation pattern might benefit from a different treatment. In fact, higher BPD symptom severity has predicted superior outcomes in DBT (Seow et al., 2020) and skills-focused therapies might be more suitable for patients with personality disorders than depressed individuals. BPD severity was not directly examined in this study, but frequency and duration of DSH behaviour has been found to be associated with higher degrees of borderline pathology (Brager-Larsen et al., 2022). Of note, the DBT-A group had a higher mean frequency of DSH as well as BPD traits at baseline. Since, in the present study, shorter duration of DSH was associated with less favourable DSH outcomes which could suggest that the treatments offered in this trial were less suitable to patients with less prominent BPD pathology.

To our knowledge, this is the first study finding early DSH response and long-term ED reductions related to DBT-A, suggesting that patients not only reduce problem behaviour, but also improve emotion regulation as they enter adulthood. This is in line with DBT studies in both adults (Axelrod et al., 2011) and adolescents (Asarnow et al., 2021) that support emotion regulation as a mechanism of change. Although our findings cannot demonstrate such an effect, they are consistent with this possibility. Adolescence constitutes a critical phase in emotion regulation development, and the association between DBT-A and long-term ED reduction suggests a potential important role of early treatment that targets ED directly. This could have long-lasting implications given ED’s assumed role as a transdiagnostic etiopathogenic factor (Gross, 2014). Nevertheless, DBT-A’s superior effect on emotion dysregulation was moderated by DSH duration. Given that longer DSH duration has been associated with more severe DSH behaviour (Brager-Larsen et al., 2022), these results suggest that DSH severity might affect ED and DSH outcomes differently.

However, emotion regulation is a complex process still under development during adolescence. Therefore, caution is warranted in interpreting ED changes as treatment-related considering the 3-year span. Indeed, it makes more sense to consider therapy with potential to influence and pivot emotion regulation development. Whether this impact can predict adult psychopathology remains unanswered and warrants studies with extended follow-up intervals. Moreover, although most participants improved, a notable proportion continued with DSH. Thus, more research is needed to further explore patient and treatment characteristics associated with response patterns. Taken together, the results supported our hypothesis of DBT-A being associated with more favourable outcomes. In addition, they supported the hypothesized predictive values of depressive symptoms and DSH duration in DSH, but not ED, response. Finally, not only did our study highlight the main effect of DBT-A at the group level, but also that earlier response across the whole sample implied an average reduction of 20 DSH yearly episodes per participant.

### Clinical Implications

DSH reduction is an important treatment target, as both a risk factor for suicide attempts (Ribeiro et al., 2016) and maintaining of emotion dysregulation - which is predictive of adult psychopathology (Young et al., 2019). Adolescents with lower levels of depression and longer DSH histories had higher probability of earlier treatment response in this study, which is relevant for clinicians’ treatment planning. Timing of treatment response, and its associated benefits as shown in this study, can have far-reaching implications for youth that struggle with DSH and their families. The World Health Organization included DSH in the top five causes of disability and burden of disease in youth worldwide (World Health Organization, 2014). Our study illustrated benefits of early, specialized treatment in reducing DSH, which have potential to impact adolescents’ burden of disease and suicide risk. Arguably, faster treatment response could enhance treatment retention, increase hope and reduce drop-out. Thus, these findings are relevant for treatment providers, adding to existing evidence of DBT-A as effective for this group (Kothgassner et al., 2021). Although most participants achieved DSH remission over a 3-year-period, the difference in *when* remission occurred shows the potential impact of specialized treatment in adolescence, as responders were spared a mean of 61 DSH episodes over 3 years. Considering that adolescent years constitutes an important phase in identity, personality and emotional development with long-lasting consequences for health, quality of life and functioning - this potential impact is striking. Moreover, compared with other BPD-specific treatments, DBT-A is relatively short and cost-effective (Haga et al., 2018). Apparently, shorter versions of DBT are as effective as lengthier programs, implicating potential to improve treatment accessibility (McMain et al., 2022). Certainly, early response in an already short-framed therapy enables adolescents to spend more time building meaningful lives and less as psychiatric patients.

### Strengths and Limitations

The experimental, prospective design in a community context enabled us to study trajectories over time and strengthens the results’ external validity. Low attrition rate combined with ITT principles reduced the risk of attrition bias. We were also able to show how effective treatment can prevent DSH episodes, across treatment conditions, in a quantifiable way. However, there are also several limitations: First, the small sample size calls for caution in interpretation, and limited the number of included predictor variables due to power considerations. In addition, it reduced the number of classes that would be meaningful to analyse in the LCA, and our choice of favouring parsimony over complexity in the modelling entailed a risk of underfitting, which might come at the expense of clinically meaningful nuance. It is important to bear in mind that our labelling of early and late responders is purely empirical and not relying on any previously acknowledged categorization. Second, as ED was not measured directly after treatment, inferences about temporal relationships between treatment and outcome were impeded. Additionally, our ED proxy variable is not formally validated, and predominately measured affective features of emotion dysregulation, while placing less emphasis on other ED aspects compared to the Difficulties in Emotion Regulation Scale (Gratz & Roemer, 2004). ED is a dynamic, multifaceted phenomenon that ideally should be measured in real-time studies (Burke et al., 2017). Third, although DSH prevalence generally was low 1.6year post-treatment, we could not discern remission during the last month of treatment or shortly thereafter (i.e. in response to the treatment) from later remission due to other factors. Finally, despite broad inclusion criteria where patients were largely representative of DBT-A’s target group, most participants were female and of Norwegian ethnicity, which impedes generalization to male and ethnic minority patients.

## Conclusions

This study contributes to the existing literature supporting DBT-A as useful for adolescents with borderline traits, both through earlier DSH reduction and improved emotion regulation. Lower levels of depression and longer DSH duration predicted treatment response, however DBT-A was the strongest predictor of favourable outcomes, which indicates that specialized treatment in adolescence can influence emotion regulation development in the long-term. Future studies with longer follow-up intervals are warranted to examine whether the reduced burden of disease in adolescence might impact psychopathology and functioning in adulthood.

## Appendix

### Abbreviations

DSH: deliberate self-harm
ED: emotion dysregulation
DBT-A: Dialectical Behavioural Therapy
EUC: Enhanced Usual Care
